# Neurodynamics and connectivity during facial fear perception: The role of threat exposure and signal congruity

**DOI:** 10.1038/s41598-018-20509-8

**Published:** 2018-02-09

**Authors:** Cody A. Cushing, Hee Yeon Im, Reginald B. Adams, Noreen Ward, Daniel N. Albohn, Troy G. Steiner, Kestutis Kveraga

**Affiliations:** 1 Athinoula A. Martinos Center for Biomedical Imaging, Massachusetts General Hospital, Charlestown, MA USA; 2 Department of Radiology, Harvard Medical School, Boston, MA USA; 3 Department of Psychology, The Pennsylvania State University, University Park, PA USA

## Abstract

Fearful faces convey threat cues whose meaning is contextualized by eye gaze: While averted gaze is congruent with facial fear (both signal avoidance), direct gaze (an approach signal) is incongruent with it. We have previously shown using fMRI that the amygdala is engaged more strongly by fear with averted gaze during brief exposures. However, the amygdala also responds more to fear with direct gaze during longer exposures. Here we examined previously unexplored brain oscillatory responses to characterize the neurodynamics and connectivity during brief (~250 ms) and longer (~883 ms) exposures of fearful faces with direct or averted eye gaze. We performed two experiments: one replicating the exposure time by gaze direction interaction in fMRI (N = 23), and another where we confirmed greater early phase locking to averted-gaze fear (congruent threat signal) with MEG (N = 60) in a network of face processing regions, regardless of exposure duration. Phase locking to direct-gaze fear (incongruent threat signal) then increased significantly for brief exposures at ~350 ms, and at ~700 ms for longer exposures. Our results characterize the stages of congruent and incongruent facial threat signal processing and show that stimulus exposure strongly affects the onset and duration of these stages.

## Introduction

When we look at a face, we can glean a wealth of information, such as age, sex, health, affective state, and attentional focus. The latter two signals are typically, but not exclusively, carried by emotional expression and eye gaze direction, respectively. Depending on the emotional expression and gaze, we can recognize how happy, angry, or fearful a person is, and infer the source or target of that emotion^1^. In an initial examination of the interaction between eye gaze and facial emotion, Adams and colleagues introduced the “shared signal hypothesis”^2–4^. This hypothesis predicts that there is facilitation of affective processing for combinations of emotional expression and eye gaze that share a congruent, matching signal for approach-avoidance behavior. In support of this hypothesis, using speeded reaction time tasks and self-reported intensity of emotion perceived, Adams and Kleck^3,4^ found that direct gaze facilitated processing efficiency and accuracy, and increased the perceived emotional intensity of approach-oriented emotions (e.g., anger and joy). Conversely, averted gaze facilitated perception of avoidance-oriented emotions (e.g., fear and sadness). Several other groups have now also found similar results, including a replication by Sander *et al*.^5^ using dynamic threat displays, another using a diffusion model of decision making and reaction time^6^, and another examining effects on reflexive orienting to threat^7^. Perhaps the most compelling behavioral replication of this effect was a study by Milders and colleagues^8^, who found that direct-gaze anger and averted-gaze fear were detected more readily in an attentional blink paradigm compared to averted-gaze anger and direct-gaze fear, suggesting that congruent pairings (i.e., shared signals) of gaze and emotion attract more preconscious attentional awareness. Similar interaction effects have been found at the neural level as well, including in several fMRI studies looking at amygdala responses to different gaze directions by threat displays, including our own (see too^9–11^).

Some have also suggested that gaze influences the ambiguity surrounding the source of threat. Whalen and colleagues, for instance, hypothesized that amygdala activation is directly proportional to the amount of ambiguity that surrounds the source of a perceived threat^12^, which suggests that direct-gaze fear is a more ambiguous signal than direct-gaze anger. Anger signals both the source of the threat and where it is directed. In the case of fear, the observer knows there is a threat, but direct eye gaze does not indicate the source of the threat - unless it is the observer. Thus, averted eye gaze is more informative in resolving the source of threat for fear, and direct gaze is more informative for anger. In both of these accounts of threat-related ambiguity (shared signals and source of threat detection), direct-gaze fear is considered a more ambiguous combination of cues than averted-gaze fear.

Initial efforts to study the neural underpinnings of the perception of these compound threat cues revealed greater amygdala activation in response to incongruent compound threat cues, specifically fearful faces with a direct gaze and anger faces with averted gaze^13,14^. Some follow-up studies, however, have found the opposite interaction: fear with averted eye gaze evoked higher amygdala activation^9,10^. To address this, Adams *et al*.^15^ proposed that presentation duration might help explain these differences. We hypothesized that brief presentations trigger more *reflexive* processing, which is thought to be preferentially tuned to congruent threat cues (averted-gaze fear), and longer presentations engage more r*eflective* processing of the less salient, incongruent threat cues (direct-gaze fear, see e.g.^16–18^ for discussions of *reflexive* vs. *reflective* processing). Indeed, previous studies finding stronger amygdala activation in response to averted-gaze fear used relatively brief stimulus exposure times (e.g.^9^ used 300 ms stimulus durations), whereas those reporting higher amygdala activation in response to direct-gaze fear had longer stimulus exposure times (e.g.,^14,19^ used 2 s and 1 s stimulus durations, respectively). Adams *et al*.^15^ put this hypothesis to the test in the context of three studies using fMRI, with varying presentation parameters during a constant 1.5 s trial. In a direct comparison, this work revealed that amygdala responses were enhanced for fearful faces coupled with averted gaze, when rapidly presented (300 ms), and to fearful faces coupled with direct eye gaze, when presented for a sustained duration (1 s).

## The Current Work

The primary goal of the present study was to elucidate the fine-grained neural dynamics of this previously observed response by replicating and extending this work using magnetoencephalography (MEG) to record neural activity in response to brief (250 ms) and longer (883 ms) presentations of fearful faces with direct and averted gaze. MEG allows us to elucidate not only the temporal evolution of neural activity, but also frequency-specific oscillatory activity in response to the stimulus, including highly temporally resolved interregional connectivity patterns during perception of these compound threat cues from the face. We utilized source localization to obtain good spatial resolution to identify the temporally sensitive contributions of key brain regions in the extended face processing network: the Fusiform Face Area (FFA), Periamygdaloid Cortex (PAC), posterior superior temporal sulcus (pSTS), and orbitofrontal cortex (OFC), as well as the earliest cortical visual region V1. These regions are well known to be involved in either face perception and social communication, or gaze perception, if not both (e.g.,^20–24^). STS in general has been shown to be sensitive to gaze^25^, as has the fusiform gyrus^26^. In addition, both have been shown to be sensitive to facial expression^27^. The STS and OFC are also implicated as nodes in the proposed “social brain” consisting of amygdala, OFC, and STS^28^. The posterior portion of STS also has been implicated as specializing in inferring intentionality from social cues^29,30^. Whether or not amygdala activity can be source localized from MEG data is actively debated in the MEG literature. However, there is accumulating evidence now that MEG activity can indeed be localized to the subcortical nuclei of the amygdala^31–35^, but supporting or advancing this claim is not the goal of our manuscript. Periamygdaloid cortex (PAC) is heavily involved in conveying inputs and outputs of the deeper amygdala nuclei, the contralateral PAC, as well many other cortical regions^36,37^. While we cannot be certain which of the amygdala nuclei the activity is coming from (a situation similar to all but the highest resolution fMRI studies), given the reliable activation of the amygdala in all of the previous studies using this paradigm it is probable that at least some of the activity may arise in the subcortical nuclei of the amygdala.

To truly understand how a network of brain regions responds to a given task, it is necessary to not only look at the response of each region individually, but to also examine how the regions interact^38^. Thus, we sought to characterize the phase locking between regions in the present work as a measure of functional connectivity within our extended face processing network. This approach allows us to build a more complete picture of the neurodynamics at play in the perception of facial threat cues. Examination of phase locking across trials within a region provides estimates of variance across trials that is not directly available in typical evoked response analyses while also having the benefit of being more robust against artifacts^39^. To examine the frequency-specific responses in our ROIs and assess functional connectivity between them, we computed phase-locking estimates in and between the evoked responses of these regions. Phase locking (in which the magnitude of oscillatory activity is normalized) was chosen over power analyses (a magnitude-dependent measure of synchronized neuronal firing) due to the fact that phase locking is by nature more informative of the timing of activity within a region as well as the timing of functional connectivity between regions^40^. Phase locking is also thought to be a more trustworthy measure of higher-level functions^41^. Oscillations in α-band (8–13 Hz) have been implicated as being involved in task selection or disengagement from the task^42–45^ while β-band (13–30 Hz) activity is indicative of active cognitive processing^46–49^. By examining activity in these bands both locally and between regions, we sought to elucidate the neurodynamics underlying the differential processing of threat cues portrayed by direct and averted gaze on a fearful face observed by Adams *et al*.^15^ when exposure duration is manipulated.

We were primarily interested in characterizing how exposure duration within a constant time frame influences threat processing as a function of threat cue congruity associated with direct or averted gaze, previously found to evoke differential activation using fMRI^15^. An adaptive response to threat cues would be a combination of reflexive and reflective processes that enables appropriate and timely responses to clear threat signals (e.g., fleeing an attacker) while also inhibiting context-inappropriate or maladaptive responses to more ambiguous threat cues (e.g., fleeing from someone who is seeking help). Temporally, initial reflexive processing has been found to begin as early as 50–100 ms, becoming fully engaged by 300 ms, while intentional responses have been found as early as 500–700 ms^50–53^. Thus, we predicted that the initial response would be stronger to averted-gaze fear within this timeframe, in agreement with the findings of Milders *et al*.^8^, which found that the congruent averted-gaze fear signal attracted more preconscious attention than direct-gaze fear. On the other hand, we predicted that direct-gaze fear would evoke a stronger secondary response indicative of reflective analysis to resolve the conflicting signals in the incongruent cue. Moreover, we hypothesized that this late-arising reflective response would be greater during longer stimulus exposures, as suggested by the fMRI results^14,15,19,54^.

Finally, we also wanted to test whether the effects reported in van der Zwaag *et al*.^54^ and Adams *et al*.^15^ can still be evoked with rapid switching of conditions in an event-related design (both direct and averted-gaze fear faces, and brief/longer exposure durations), rather than state-dependent, which could be the case for previous work employing block designs. Therefore, we employed a rapid event-related design in MEG and also scanned a separate cohort in fMRI using an identical paradigm. An additional motivation was to compare the periamygdaloid complex activation in MEG to the fMRI results to test whether the PAC activity we found in MEG is at least broadly comparable to the BOLD activity in fMRI. While BOLD activity is unable to capture the fine-grained temporal dynamics present in the MEG signal, the overall activation differences might be comparable^55^.

## Methods and Materials

### Experiment 1

#### Participants

Total 28 undergraduate students (22 females), mean age (s.d.) = 18.75 (1.73), participated in Experiment 1. All the participants had normal color vision and normal or corrected-to-normal visual acuity. Their informed written consent was obtained according to the procedures of the Institutional Review Board at the Pennsylvania State University. The participants received partial course credit for their participation. This research was performed in accordance with the guidelines and regulations set forth in the Declaration of Helsinki, and was approved by the Institutional Review Board of the Pennsylvania State University.

#### Stimuli

**Figure 1:**
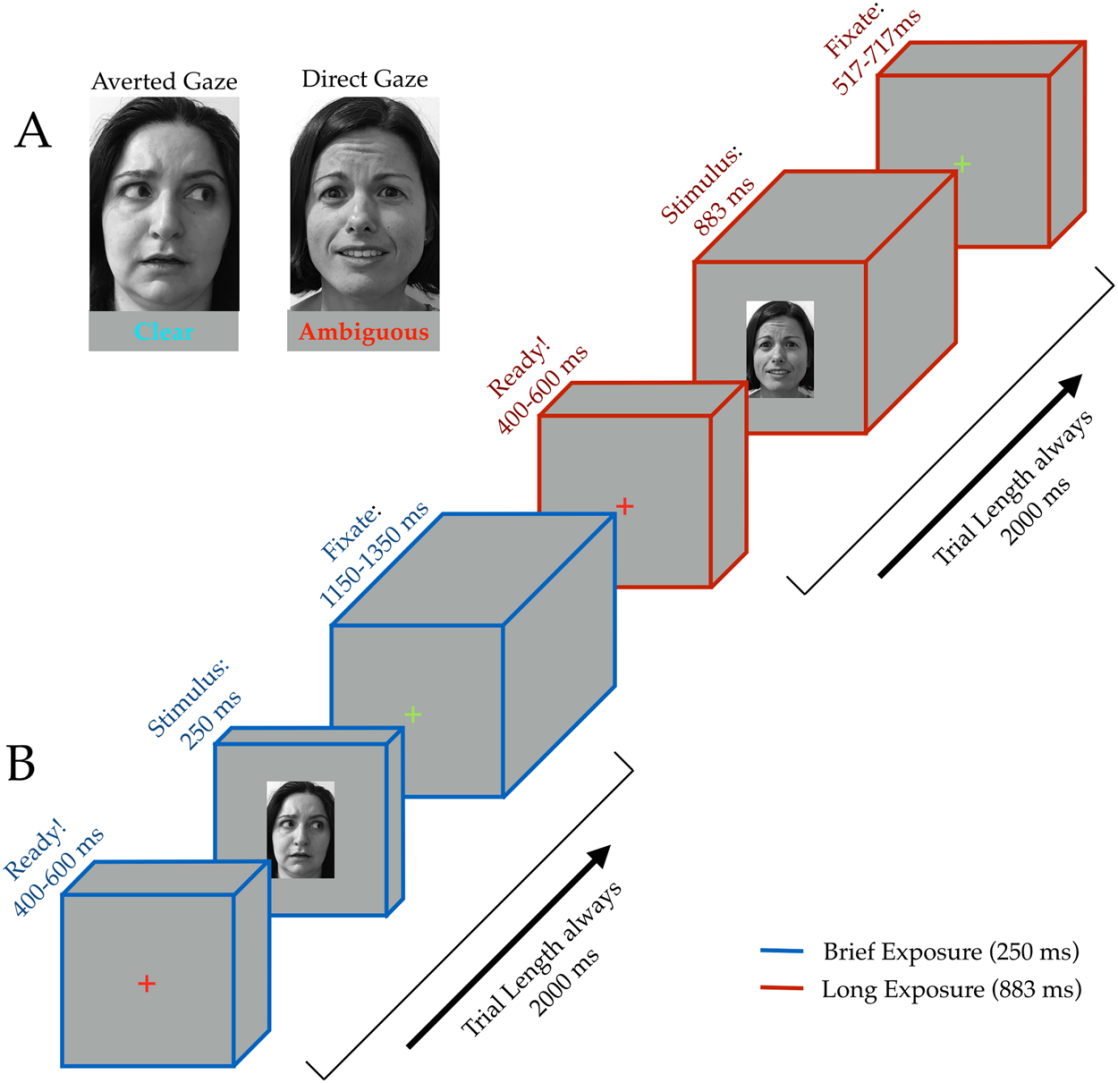
Stimuli and Task Design. (**A**) Examples of fearful expression by eye gaze pairings. All faces shown to participants in the actual experiment were taken from the NimStim or Ekman databases (not shown here due to publishing permissions) and displayed a fearful expression with either direct or averted gaze. Participants were instructed to make a button response if the stimulus was a non-face object, ensuring attentive viewing of all faces. (**B**) Sequence depicting one each of both brief (blue cuboids) and long (red cuboids) exposure trial types. Trials are always 2000 ms: 400–600 ms of a red fixation cross signifying trial start, followed by 250 ms or 883 ms of stimulus (corresponding to trial type), concluding with either 1150–1350 ms or 517–717 ms of a green fixation cross, dependent upon trial type. End-trial jitter is inversely timed with pre-trial jitter such that trial lengths are kept a constant 2000 ms.

The face stimuli in this experiment were identical to those used in the three studies reported in Adams *et al*.^15^, with eight models (four female) from the Pictures of Facial Affect^56^ and eight models (four female) from the NimStim Emotional Face Stimuli database^57^, all displaying a fearful expression with either a direct gaze or averted gaze (Fig. 1A). Each model had 6 stimuli unique to their identity: one with direct gaze, one with leftward averted gaze, one with rightward averted gaze, and then the mirror image of each of these iterations. This resulted in 96 unique face stimuli used in the experiment. Images of ordinary household objects were also used to ensure observer attention and required a response. Stimuli were presented with Psychtoolbox^58,59^ in Matlab (Mathworks Inc., Natick, MA). Stimuli were projected onto a screen at the head of the bore and viewed via an angled mirror attached to the head coil subtending approximately 5.72° horizontally and 7.77° vertically of visual angle.

#### Task Design

We employed an event-related design while keeping presentation parameters as similar to Adams *et al*.^15^ as possible within such a design. Each participant viewed 320 trials over the four runs that were randomly, evenly split into brief (15 frames at a refresh rate of 16.67 ms totaling 250 ms) and longer stimulus (53 frames at a refresh rate of 16.67 ms totaling 883 ms) durations to get 160 trials for each presentation duration (128 of which were faces). Each trial lasted 2 seconds, beginning with a randomized 400–600 ms of attending to a central red fixation cross. The stimulus (either a face or an object) was then presented for either 250 ms or 883 ms, dependent upon trial type, followed by a green fixation cross for the remainder of the trial (ranging from 517–1350 ms) before switching back to red to signify the start of a new trial (Fig. 1B). Participants were instructed only to make a manual response via a button box if the stimulus was a non-face object. This task would allow us to ensure that participants pay attention to faces without explicitly labeling the emotion displayed, as previous studies have shown that the act of emotion labeling changes neural responsivity to the emotional expression^60^.

#### fMRI Data Acquisition and Analysis

fMRI images of brain activity were acquired using a 3 T scanner (Siemens Magnetom Prisma) located at The Pennsylvania State University Social, Life, and Engineering Sciences Imaging Center. High-resolution anatomical MRI data were acquired using T1-weighted images for the reconstruction of each subject’s cortical surface (TR = 2300 ms, TE = 2.28 ms, flip angle = 8°, FoV = 256 × 256 mm^2^, slice thickness = 1 mm, sagittal orientation). The functional scans were acquired using gradient-echo EPI with a TR of 2000 ms, TE of 28 ms, flip angle of 52° and 64 interleaved slices (3 × 3 × 2 mm resolution). Scanning parameters were optimized by manual shimming of the gradients to fit the brain anatomy of each subject, and tilting the slice prescription anteriorly 20–30° up from the AC-PC line as described in the previous studies^61,62^ to improve signal and minimize susceptibility artifacts in the brain regions susceptible to signal dropout, such as OFC^63^. We acquired 456 functional volumes per subject in four functional runs, and the sequence of trials was optimized for hemodynamic response estimation efficiency using *optseq. 2* software (https://surfer.nmr.mgh.harvard.edu/optseq/).

The acquired fMRI data were analyzed using SPM8 (Wellcome Department of Cognitive Neurology, http://www.fil.ion.ucl.ac.uk/spm/software/spm8/). The functional images were corrected for differences in slice timing, realigned, corrected for movement-related artifacts, coregistered with each participant’s anatomical data, normalized to the Montreal Neurological Institute template, and spatially smoothed using an isotropic 8-mm full width half-maximum (FWHM) Gaussian kernel. ArtRepair software was used to correct for excessive movement (http://spnl.stanford.edu/tools/ArtRepair/ArtRepair.htm), and outliers due to movement or signal from preprocessed files, using thresholds of 3 s.d. from the mean, 0.75 mm for translation and 0.02 radians rotation, were removed from the data sets^64^. Data of five participants among the 28 participants were unusable with ArtRepair identifying >75% of scans as outliers. Therefore, only the remaining 23 participants (19 females and 4 males, mean age (s.d.) = 19.13 (1.36)) were included for further fMRI analyses.

Subject-specific contrasts were estimated using a fixed-effects model. These contrast images were used to obtain subject-specific estimates for each effect. For group analysis, these estimates were then entered into a second-level analysis treating participants as a random effect, using one-sample t-tests at each voxel. We computed contrasts between brief and longer exposures within each Threat type (i.e., brief averted-gaze fear vs. longer averted-gaze fear, brief direct-gaze fear vs. longer direct-gaze fear) and between averted-gaze fear and direct-gaze fear within each exposure duration, separately. The full lists of these whole brain activations are shown in Tables 1 through 3, thresholded at *p* < 0.001 (*t* > 3.505) and a minimal cluster size of 10 voxels. These parameters are more conservative than those that have been argued to optimally balance between Type 1 and Type 2 errors (^65^: height *p* < 0.005, uncorrected, extent: 10 voxels; see also^15,46^). Although cluster-extent based thresholding has become the most popular approach to dealing with multiple comparisons (e.g.^66–68^), we chose to report uncorrected *p-*values using these thresholds for two reasons. First, cluster-extent based thresholding has low spatial specificity when clusters are large^66,68,69^, such that *p*-values of activation at a specific location or an anatomical area within the cluster are not specifically determined. Second, we sought to replicate the prior findings by Adams *et al*.^15^, in which uncorrected *p*-values were reported at threshold of *p* < 0.005 and k = 10. Thus, reporting uncorrected *p*-values using these parameters would allow us to ensure spatial specificity of the statistical significance of activation at the areas that have also been reported previously in Adams *et al*.^15^.

**Table 1:** Regions showing increased activation in Experiment 1 in brief- minus longer-exposure, and longer- minus brief-exposure contrasts for averted-gaze fear faces (clear threat) and direct-gaze fear faces (ambiguous threat) (height: *p* < 0.001 (*t*(22) > 3.505), extent = 10 voxels).

Region Label	t-value	Extent	MNI coordinates		
			x	y	z
***Averted gaze***					
*Brief - Long*					
L Brainstem	4.797	33	−3	−10	−26
R Lingual gyrus	3.524	14	30	−67	8
R Amygdala	4.574	11	27	2	−26
L Frontal cortex (Frontal eye fields, BA8)	4.498	31	−24	17	42
L Posterior superior temporal sulcus	4.397	17	−42	−49	14
L Parahippocampal cortex	4.289	16	−24	−7	−30
L Superior frontal gyrus	4.232	15	−21	−10	58
L Inferior parietal lobule	4.162	73	−30	−55	38
R Inferior frontal gyrus	4.099	32	42	11	30
Long - Brief					
L Visual cortex (BA 18)	6.314	221	−18	−100	6
R Visual cortex (BA 17)	6.259	473	15	−97	2
R Visual cortex (BA 18)	3.871	—	33	−94	14
***Direct gaze***					
*Brief - Long*					
None					
*Long - Brief*					
R Visual cortex (BA 18)	3.566	6496	18	−79	4
	9.223	—	21	−94	−8
L Fusiform gyrus	7.34	—	−27	−79	−10
R Posterior cingulate cortex	8.005	45	−12	−43	26
R Fusiform gyrus	5.315	35	27	2	−36
L Intraparietal sulcus	4.951	57	−33	−82	40
R Middle Cingulate Cortex	4.784	26	9	−4	40
L Temporal pole	4.724	43	−39	14	−32
L Fusiform gyrus	4.158	23	−36	−22	−20
R Cerebelum	4.131	41	12	−52	−36
R Middle temporal gyrus	4.072	15	57	−7	−20
R Postcentral gyrus	3.908	12	48	−25	40
L Lateral orbitofrontal cortex	3.89	11	−54	29	−10

— indicates that this cluster is part of a larger cluster immediately above.

**Table 2:** Regions showing increased activation in Experiment 1 in averted-gaze (clear threat) minus direct-gaze (ambiguous threat) and direct-gaze minus averted-gaze contrasts during brief and long exposures (height: *p* < 0.001 (*t*(22) > 3.505), extent = 10 voxels).

Region Label	t-value	Extent	MNI coordinates		
			x	y	z
***Brief Exposure***					
*Averted gaze - Direct gaze*					
R Cerebelum	6.019	102	33	−43	−24
L Cerebelum	7.302	533	12	−40	−28
	6.775	—	−27	−46	−26
	4.188	533	−6	−61	−16
R Inferior frontal gyrus	3.871	11	45	11	30
L Inferior frontal gyrus	6.245	113	−39	2	34
L Inferior temporal gyrus	4.492	121	−33	−1	−30
R Retrosplenial cortex	5.238	327	27	−49	10
R Visual cortex	4.291	—	24	−76	10
	4.126	—	15	−91	−6
L Visual cortex	4.095	82	−21	−76	4
L Parahippocampal cortex	4.464	13	−15	−31	−2
L Medial temporal pole	4.327	15	−36	14	−32
R Postcentral gyrus	4.325	40	24	−46	58
R Middle cingulate cortex	4.275	18	3	−10	36
L Lingual gyrus	4.2	82	−27	−55	2
L Precentral gyrus	3.531	12	−63	−1	30
R Amygdala	3.53	19	21	−1	−24
*Direct gaze - Averted gaze*					
None					
***Long Exposure***					
*Averted gaze - Direct gaze*					
None					
*Direct gaze - Averted gaze*					
L Cerebelum	5.57	241	−24	−91	−24
R Visual cortex	5.56	137	15	−52	−12
	4.918	180	30	−88	−20
L Visual cortex	5.539	195	−15	−49	−12
L Lingual gyrus	4.363	—	−12	−64	6
R Fusiform gyrus	4.049	12	39	−16	−32
L Fusiform gyrus	5.015	82	−33	−13	−40
R Temporal pole	4.063	12	33	−1	−42
L Temporal pole	4.949	82	−30	8	−46
R Parahippocampal cortex	4.756	33	30	−34	−12
	4.109	11	21	−1	−34
L Cuneus	4.733	77	−18	−76	30
L Prefrontal cortex	4.702	27	−18	62	20
L Posterior cingulate cortex	4.626	29	−12	−40	36
L Angular gyrus	4.352	79	−39	−64	38
L Superior temporal sulcus	4.329	37	−48	−28	−4
R Intraparietal lobule	4.244	131	42	−52	38
L Posterior superior temporal sulcus	4.193	37	−42	58	18
R Inferior frontal gyrus	4.066	29	51	35	30
L Inferior frontal gyrus	4.103	46	−57	11	36
R Inferior temporal gyrus	4.088	63	66	−31	−20
R Prefrontal cortex	3.998	17	9	65	26
L Superior temporal gyrus	3.925	12	−60	−13	−18
R Superior frontal gyrus	3.905	20	27	62	24
L Amygdala	3.857	13	−18	2	−22

— indicates that this cluster is part of a larger cluster immediately above.

**Table 3:** Regions showing increased activation in Experiment 1 in the interaction between stimulus exposure and gaze direction of fearful faces, measured by the contrast (Brief averted gaze & Long direct gaze) – (Long averted gaze & Short direct gaze) (height: *p < *0.001 (*t*(22) > 3.505), extent = 10 voxels).

Region Label	t-value	Extent	MNI coordinates		
			x	y	z
***Interaction***					
*(Brief averted gaze & Long direct gaze) – (Long averted gaze & Short direct gaze)*					
L Cerebelum	6.944	4804	−15	−52	−16
R Cerebelum	6.526	4804	24	−85	−14
R Cerebelum	6.523	—	15	−49	−14
L Amygdala	6.111	182	−21	−1	−26
L Medial Temporal Pole	4.362	182	−39	14	−32
R Amygdala	5.407	107	27	2	−26
L Inferior frontal gyrus	5.353	185	−48	5	32
L Inferior temporal gyrus	5.284	114	−66	−16	−28
L Pallidum	5.255	68	−9	−1	2
R Middle cingulate cortex	5.126	24	6	−4	38
R Inferior fronta gyrus	5.038	205	57	14	36
R Precentral gyrus	3.912	205	45	−4	36
L Superior temporal sulcus	5.010	19	−39	−28	−4
R Cerebelum	4.830	56	42	−55	−42
L Superior temporal sulcus	4.769	33	−51	−10	−18
R Inferior temporal gyrus	4.734	56	63	−13	−34
L Cerebelum	4.638	29	−27	−52	−44
L Precentral gyrus	4.581	11	−36	−13	44
R Middle frontal gyrus	4.580	49	51	32	26
R Angular gyrus	4.536	32	39	−49	36
R Middle temporal gyrus	4.402	49	69	−22	−20
L Posterior cingulate cortex	4.378	28	−12	−40	34
L Putamen	4.318	20	−27	−13	2
L Inferior temporal gyrus	4.290	15	−57	−1	−36
L Orbitofrontal cortex	4.071	13	−51	41	−12
L Posterior superior temporal sulcus	4.044	65	−45	−37	6
L Hippocampus	3.991	11	−27	−19	−18
R Premotor cortex	3.944	29	36	−16	42
R Fusiform gyrus	3.883	14	30	−31	−16

— indicates that this cluster is part of a larger cluster immediately above.

**Figure 2:**
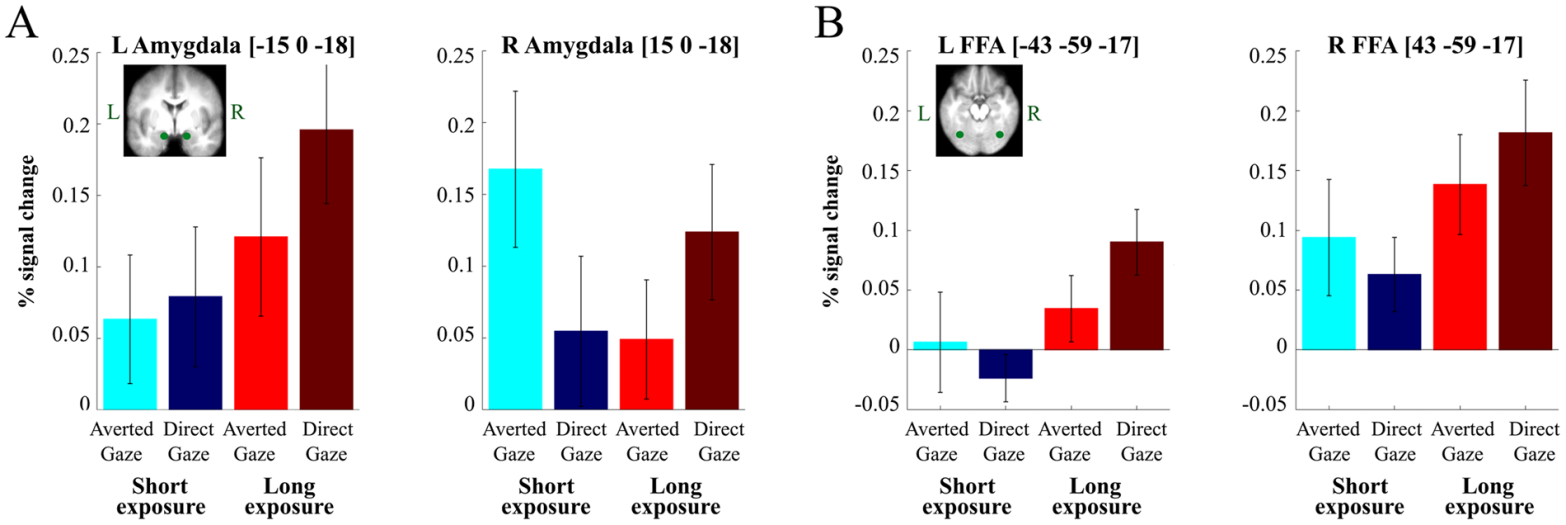
Results from Experiment 1 (fMRI study). (**A**) Shows the percent change in the BOLD signal in bilateral amygdala (left = left hemisphere, right = right hemisphere) in response to brief (blue colors) and long (red colors) to clear threat cues (brighter colors) and ambiguous threat cues (darker colors). (**B**) Shows the percent change in the BOLD signal in bilateral Fusiform Face Area (FFA) (left = left hemisphere, right = right hemisphere) in response to brief (blue colors) and long (red colors) to clear threat cues (brighter colors) and ambiguous threat cues (darker colors). Note for both (**A**) and (**B**), that a clear threat cue is represented by a fearful face with averted eye-gaze and an ambiguous threat cue is represented by a fearful face with direct eye-gaze.

For the regions of interest (ROI) analyses, we extracted the BOLD activity from our *a priori* ROIs: the amygdala, FFA, OFC, and pSTS. We defined another contrast between all the visual stimulation trials (the brief and longer exposures of averted-gaze fear and direct-gaze fear) vs. Null trials. From this contrast, we extracted the percent signal change in our ROIs for all the four conditions using the rfxplot toolbox (http://rfxplot.sourceforge.net) for SPM. We identified the [x y z] coordinate for each of our ROIs (the MNI coordinates are shown in Fig. 2) then defined a 6 mm sphere around it. The coordinates for Amygdala, FFA, and OFC were determined based on the previous research that reported the involvement of these regions in processing emotional facial expression (e.g.,^14,15^). Using the rfxplot toolbox in SPM8, we extracted all the voxels from each individual participant’s functional data within that sphere. The extracted percent signal change for each of the four trial conditions was subjected to a two-way repeated-measures ANOVA with the factors of Exposure duration (2 levels: brief (250 ms) vs. longer (883 ms)) and Threat type (2 levels: averted-gaze fear vs. direct-gaze fear), separately for each of the ROIs. In addition, due to our previous findings of left amygdala being specifically sensitive to direct-gaze fear (incongruent threat cue) during the longer exposures^15^, we performed a planned comparison for left hemisphere ROIs of longer exposure direct-gaze fear to all other conditions.

### Experiment 2

#### Participants

Sixty participants (40 females), mean age (s.d.) = 26.6 (6.9), with normal or corrected to normal vision completed the study for monetary compensation ($50). Potential subjects were screened via a questionnaire to make sure they were eligible for MEG recording and subsequent MRI structural scans, and had no history of mental illness or use of psychoactive medication. Their informed written consent was obtained according to the protocol approved by the Institutional Review Board of MGH. This research was performed in accordance with the guidelines and regulations set forth in the Declaration of Helsinki, and was approved by the Institutional Review Board of the Massachusetts General Hospital.

#### Stimuli and Task Design

Stimuli and task design were identical to Experiment 1 with the exception of breaks between runs in the MEG being self-paced until the participant was ready to continue. Stimuli were rear-projected onto a translucent screen placed 160 cm from the seated participant to create a 61.5 cm × 38.5 cm display. Stimuli measured 14.1 × 19.2 cm subtending about 5.1° of visual angle horizontally and 6.9° vertically.

#### MEG Acquisition

Magnetoencephalogram recordings were obtained with a 306-channel Neuromag Vectorview whole-head system (Elekta Neuromag) with 204 planar gradiometers and 102 magnetometers enclosed in a magnetically shielded room with a shielding factor of 250,000 at 1 Hz (ImedcoAG). Four head position indicator (HPI) electrodes were affixed asymmetrically to each participant’s forehead and the mastoid processes to monitor head position in the dewar at the beginning of the recording session. Digitizer data were collected for each participant’s head on a Polhemus FastTrack 3D system within a head-coordinate frame defined by anatomical landmarks (left preauricular area, right preauricular area, and the nasion). HPI positions were marked within this frame, and 150–200 points on the scalp and the face were entered for co-registration with structural MRIs of the subject. Eye movements and blinks were monitored via 4 EOG electrodes: 2 vertical electrodes on the left eye (one placed just above the eyebrow, the other on the upper cheekbone just below the eye), and 2 horizontal electrodes (placed on either side of the head between the eye and hairline). Cardiac activity was recorded via ECG using electrodes placed on the left and right chest (2 total). All data from MEG sensors and EOG and ECG electrodes were sampled at 600.615 Hz and were band-pass filtered at 0.1–200 Hz. Recordings were stored for offline analysis.

#### Data Pre-processing and Averaging

All recordings were pre-processed and averaged using a combination of the MNE analysis package^70^ as well as MNE-Python^71^ and custom scripts in Python and Matlab. Signal-space projection was applied to the recordings in order to remove noise from external sources^72,73^. Sensors that were visibly noisy during the recording were noted by the researchers and excluded from analysis. For time course analysis, a low-pass filter of 40 Hz was applied, and recordings were epoched from 200 ms before stimulus onset to 1300 ms post-stimulus. For time-frequency analysis, no filter was applied to the data, and recordings were epoched from 500 ms before stimulus onset until 1440 ms post-stimulus onset. Rejection parameters were set at 4,000 fT/cm for gradiometers, 4000 fT for magnetometers, and 800 uV for EOG. Any epoch where any of these limits were exceeded was excluded from further analysis. A further data quality inspection was performed during preprocessing and any noisy or flat channels that were not picked up during the recording, but resulted in the rejection of 20% or more of epochs were excluded from analysis to prevent unnecessary epoch rejection. No participants were excluded due to excessive trial rejection. The lowest number of trials entered into the analyses for a condition for any subject was 51 trials. There were no significant differences between conditions for trial count (all *p*’s > 0.86). We excluded trials where object images were presented from MEG analyses as they were of no experimental interest.

#### Source Localization

A structural MRI for each participant was acquired on a 1.5 T Siemens Avanto 32-channel “TIM” system. A single compartment boundary-element model was fitted to the intracranial volume of the MRI data in the form of a triangular mesh isomorphic to an icosahedron recursively divided 5 times. This model was implemented in a surface-based forward solution to restrict the sources of the MEG signal to the vertices of this triangular mesh (source space) fitted to each individual’s inflated cortical surface reconstructed using the Freesurfer analysis package^74^. Sources closer than 5 mm to the inner skull surface were omitted from the forward solution. The MRI-head coordinate transformation for each subject was supplied to the forward model by aligning the digitizer data obtained in the original recording session (see *MEG Acquisition*) with a high-resolution head surface tessellation constructed from the MRI data. The inverse operator was prepared with a loose orientation constraint (LOC) parameter of 0.2 in order to improve localization accuracy^75^. A depth-weighting coefficient of 0.8 was also set for the inverse operator to lessen the tendency of minimum-norm estimates to be localized to superficial currents in place of deep sources. Only gradiometers were used in the depth weighting process. Both gradiometers and magnetometers were used to source localize the data. MEG data were source localized onto the whole brain using a lambda2 regularization parameter based on Signal-to-Noise Ratio (SNR) equal to 1/(SNR^2^). Evoked cortical activation was quantified spatiotemporally by taking only the radial component from a 3-orientation source [x y z] at each vertex in the form of dynamic statistical parametric maps (dSPMs) based on an inverse solution regularized with an SNR of 3. These are a statistical representation of significant activity from each source per time point calculated by noise-normalization on the estimated current amplitude (MNE) of a given source according to noise covariance between sensors calculated during a baseline period of 200 ms pre-stimulus^76^. The noise covariance estimation model was selected automatically according to rank for each participant^77^. To analyze the spectral content of the neural response, MEG data were source localized on a trial-by-trial basis using the minimum-norm estimate (MNE) method with the same inverse operator regularized with an SNR of 1 due to the indiscernibility of signal and noise at the single trial level.

#### ROI Selection and Definition

Regions of interest (ROIs) were chosen based on their established roles in early visual processing, face perception, threat detection, and emotional processing: early visual cortex (V1), fusiform face area (FFA), posterior superior temporal sulcus (pSTS), periamygdaloid cortex (PAC), orbitofrontal cortex (OFC). For the MEG data analyses, the ROIs (‘labels’ in the terminology of the *mne_analyze* software) were functionally derived in each individual’s anatomical space within *a priori* anatomical constraints (automatic cortical parcellations) produced with the Freesurfer analysis package^74^, except for the PAC and pSTS as explained below. The functional label within the anatomical parcellation was derived from averaged activity from all conditions, so that the activity was independent of trial type. This enabled us to account for intersubject variability in regions like FFA, pSTS, and OFC. Functional labels were generated within the anatomical parcellation corresponding to the ROI by isolating the source-space vertex with the highest activation within the anatomical constraints as well as neighboring vertices in the source-space (also within the anatomical constraints) that reach at least 60% of the maximum activation (in dSPM values). Since no suitable *a priori* parcellation of the amygdala and surrounding periamygdaloid cortex was available, a posteriori anatomical constraints were imposed in the form of user-drawn ROIs in the Freesurfer software on the *fsaverage* inflated surface corresponding to the cortex surrounding and including the amygdala, which we will hence refer to simply as periamygdaloid cortex (PAC). The drawing of the PAC labels was tracked by linking the drawn points to be displayed on the *fsaverage* MRI volume in *tkmedit* to ensure that only the cortical surface corresponding to the amygdalae was included in the label. These anatomical constraints were then morphed to each individual’s inflated surface and used to generate functional PAC ROIs according to the preceding procedure. A similar method was used to obtain the posterior portion of STS as the *a priori* parcellation generated by Freesurfer extended beyond the true pSTS on many subjects’ cortical surfaces to inferior sulci. User-marked constraints on the *fsaverage* inflated surface were marked around STS and tracked in the *fsaverage* MRI volume. The same morphing procedure from above was used, and then the label was split into thirds. The most posterior third was then taken as each individual’s pSTS to be used as the anatomical constraint when generating the functional pSTS labels.

#### Time-Course Analysis

Time courses were produced for each ROI by averaging the activity from source-space vertices that fell within the label marked on the individual’s inflated cortical surface to be submitted to statistical analysis. The individual average activity was then further averaged across subjects in order to visualize the grand average.

#### Phase-Locking Analysis

Using modified scripts from the MNE-Python package^71^, the Phase-Locking Factor (PLF) across trials was calculated for each ROI, and Phase-Locking Value (PLV) was calculated to assess functional connectivity between two ROIs. The PLF is a number between 0 and 1 (1 representing perfect synchrony) that represents a magnitude-normalized measure of the phase angle consistency across trials for a particular time-point at a particular frequency^78^. This number was obtained by source localizing each epoch into source space using the Minimum-Norm Estimate (MNE) method with the sign of the signal preserved. Source-space MNE epochs were subjected to spectral decomposition at each time point for each frequency of interest, using a continuous wavelet transformation with a family of complex morlet wavelets containing a number of cycles equal to f/7, where f denotes the frequency of interest. This keeps the time window at each frequency identical resulting in stable temporal resolution across frequency ranges. We analyzed frequencies from 8 Hz (representing the lower limit of the α-band) to 30 Hz (representing the upper limit of the β-band). To make these results easier to interpret, and in attempt to localize effects away from spectral leakage inherent in such transformations, PLFs were analyzed in separate frequency ranges: α (8–13 Hz), β (13–30 Hz). Similarly, inter-regional connectivity was assessed with PLVs, also a magnitude-normalized measure of phase-angle consistency across trials between 2 ROIs. This was calculated with the same parameters on the same frequencies as above (8–30 Hz) and analyzed by frequency band.

#### Statistical Analyses

As this work is a direct extension of Adams *et al*.^15^, we approached this work with particular effects whose temporal properties we sought to investigate. Specifically, we knew that averted-gaze fear elicits stronger BOLD activity relative to direct-gaze fear during brief presentations and the opposite is true of longer presentations. Consequently, we performed non-parametric comparisons based on *t*-tests comparing direct vs. averted-gaze fear faces within each presentation duration to describe the temporal evolution of these opposing sensitivities. Additionally, to verify our presentation duration manipulations were functioning as intended (i.e., to perform a reality check), we compared brief vs. longer presentations within each gaze direction, also using non-parametric comparisons based on *t*-tests. All statistics were computed using non-parametric cluster-level permutation tests based on 5000 permutations with a critical alpha-value of 0.05, following Maris and Oostenveld^79^. Cluster mass was determined by summing t-values within the cluster rather than counting significant pixels/time-points. Reported *p*-values are Monte Carlo *p-*values comparing the observed cluster to a null distribution comprising the largest cluster yielded by permuted data sets. That is, the reported *p-*value is the percentage of permuted data sets that yielded a larger cluster than the actual observed cluster (e.g., *p* = 0.05 means 250 out 5000 permuted data sets yielded a larger cluster than the observed cluster).

*Time Domain*. Statistical analyses in the time domain were performed by subtracting each participant’s evoked response for 2 conditions of interest to create a contrast wave. Null distributions were built by means of a sign-flip permutation based on a one-sample t-test. Observed clusters of significant time-points whose masses were exceeded by 5 percent of or fewer clusters from the null distribution were considered significant.

*Time-Frequency Domain*. Phase-locking maps for each participant (2-dimensional images where the y-axis represents frequency and the x-axis represents time with pixel values corresponding to phase-locking factors or values) were smoothed via a Gaussian image filter with a kernel size of 5 and a sigma of 2 before being submitted to permutations and statistical analysis. Permutations were performed by shuffling condition labels for each participant, such that the condition label of each participant’s phase-locking map was randomized but no one subject ended up with both phase-locking maps (the unit of observation in this case) falling under the same condition. Both the observed and permuted statistical maps were thresholded at an alpha-level of 0.05 with 59 degrees of freedom in order to identify clusters. Observed clusters of contiguous supra-threshold time-frequency points whose masses were exceeded by 5 percent or less of clusters from the null distribution were considered significant.

*Data Availability*. All data and code used to perform analyses reported herein are available from the corresponding author at reasonable request.

## Results

### fMRI Results

Our main interest was in the amygdala responses to averted-gaze vs. direct-gaze fear (congruent vs. incongruent threat cues) and their interactions with brief vs. longer stimulus exposures. Figure 2A shows the percentage of BOLD signal change from the baseline for each of the four trial conditions (the brief and longer exposures of averted-gaze fear and direct-gaze fear) in the left and right amygdala. A two-way repeated measures ANOVA with the factors of the Exposure duration (2 levels: brief [250 ms] vs. longer [883 ms]) and the Threat type (2 levels: averted-gaze fear vs. direct-gaze fear) showed a significant main effect of exposure duration in the left amygdala (*F*(1,22) = 7.19, *p* < 0.015), such that the left amygdala (Fig. 2A, left panel) showed greater activation for longer exposure than for brief exposure of threat cues (Fig. 2A, left panel). However, neither the main effect of Threat type (*F*(1,22) = 1.564, *p* = 0.224) nor the interaction between the Exposure duration and the Threat type (*F*(1,22) = 0.496, *p* = 0.489) were statistically significant. Based on our *a priori* hypothesis that the left amygdala would be more involved in the sustained processing of the incongruent threat cue (the longer exposure of direct-gaze fear), we conducted a planned comparison to compare the longer exposure of direct-gaze fear with any other conditions and observed a marginally significant effect (*t*(22) = 1.70, *p* = 0.093).

In the right amygdala (Fig. 2A, right panel), both main effects of the Exposure duration (*F*(1,22) = 0.295, *p* = 0.593) and of the Threat type (*F*(1,22) = 0.208, *p* = 0.652) were not significant. However, the predicted interaction between Exposure duration and Threat type was significant (*F*(1,22) = 7.19, *p* < 0.015). Specifically, the right amygdala responded more strongly to the averted-gaze fear when the exposure was brief and to direct-gaze fear when the exposure was longer. These results are consistent with previous findings that indicate possible amygdala lateralization in orientation and evaluation of facial threat cues^80^.

In the left FFA (Fig. 2B, left panel), we found significantly greater activation for longer exposure over brief exposure, confirmed by a significant main effect of the Exposure duration (*F*(1,22) = 5.491, *p* < 0.03). Although a main effect of Threat type (*F*(1,22) = 0.284, *p* = 0.600) and the interaction between Exposure duration and Threat type (*F*(1,22) = 1.766, *p* = 0.198) were not statistically significant, the same planned comparison as in the left amygdala (i.e., the longer exposure of direct-gaze fear vs. other conditions) showed a significant effect (*t*(22) = 2.076, *p* < 0.05). In the right FFA (Fig. 2B, right panel), the main effect of Exposure duration was also significant (*F*(1,22) = 6.620, *p* < 0.02). Furthermore, we observed a similar activation pattern in the right FFA as in the right amygdala: The brief exposure of averted-gaze fear and the longer exposure of direct-gaze fear elicited stronger right FFA responses than their counterparts (brief exposure of direct-gaze fear and longer exposure of averted-gaze fear, respectively, see Fig. 2B, right panel). However, the interaction between Exposure duration and Threat type (*F*(1,22) = 1.221, *p* = 0.281) was not significant nor was the main effect of Threat type (*F*(1,22) = 0.029, *p* = 0.867). Thus, the left and right FFA responses somewhat resembled those in the left and right amygdala, but were weaker: Greater activation was observed for the longer exposure of direct-gaze fear in the left FFA and greater activation for the brief exposure of averted-gaze fear and the longer exposure of direct-gaze fear in the right FFA. It is also worth noting that the right FFA (Fig. 2B, right panel) showed greater levels of activation than the left FFA (Fig. 2B, left panel) in all the conditions, consistent with previous findings on the right hemisphere dominance in function of FFA^81–84^. To confirm this statistically, we conducted a separate three-way repeated measures ANOVA with additional factor of Hemisphere (left FFA versus right FFA) along with the Exposure duration and Threat type, and found a significant main effect of Hemisphere (*F*(1,22) = 7.465, *p* < 0.015).

Consistent with the ROI results, the statistical analyses on the whole brain using a univariate GLM approach showed the greater right amygdala activation for the brief exposure of averted-gaze fear, compared to the longer exposure, and the greater bilateral amygdala for the longer exposure of direct-gaze fear, compared to the brief exposure (Table 1). As shown in Table 1, we also observed that the other ROIs including the OFC, Fusiform area, V1, and the pSTS were differentially activated as a function of the Exposure duration by Threat type interaction, with greater responses for the brief exposure of averted-gaze fear and the longer exposure of direct-gaze fear. Supporting this finding at the whole brain level, there is unanimously stronger activity to averted-gaze fear relative to direct-gaze fear during brief exposures and to direct-gaze fear compared to averted-gaze fear during longer exposures (see Table 2).

### MEG Results

#### Effects of Exposure Duration: Time Courses

**Figure 3:**
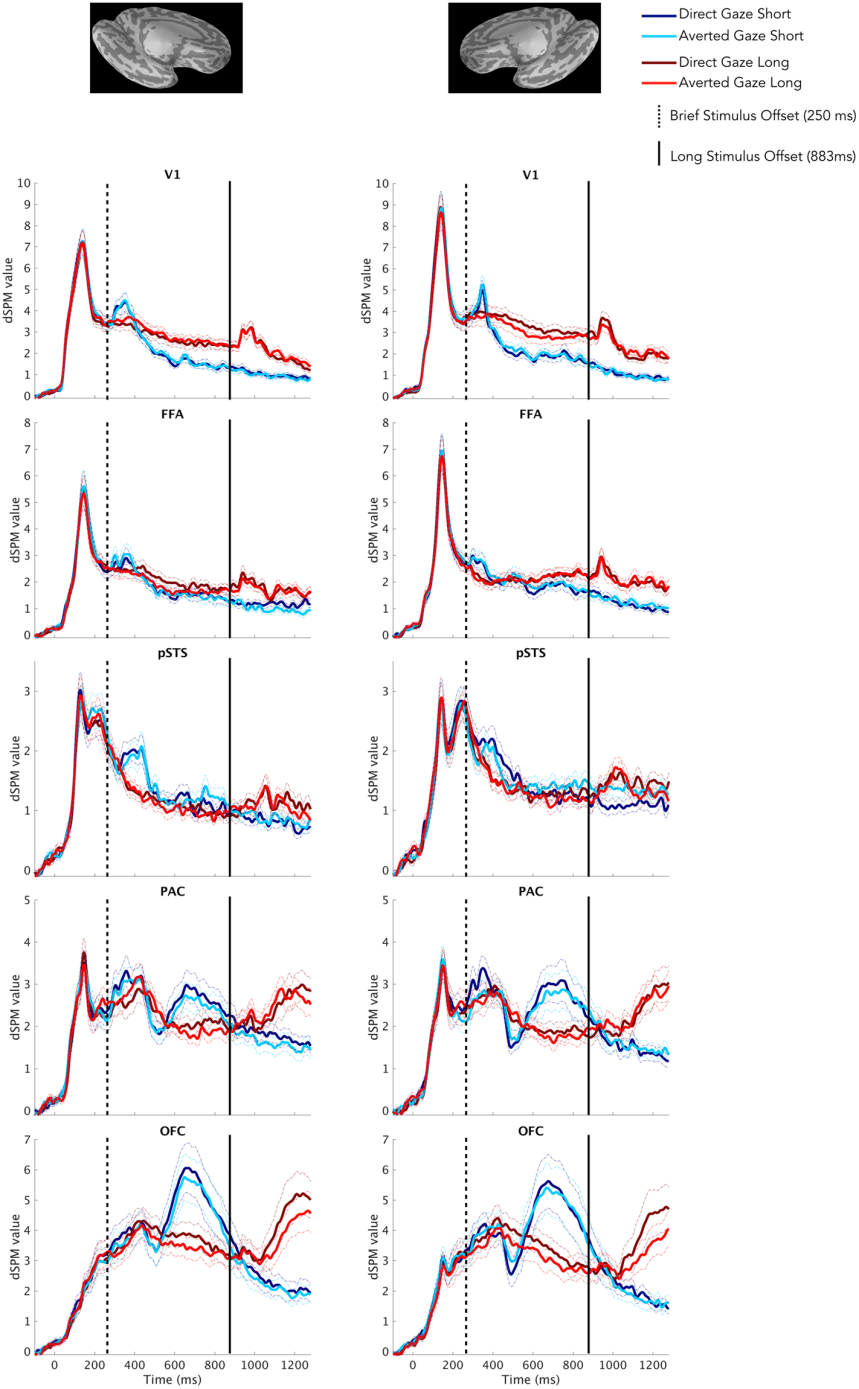
Time courses of activation within Regions of Interest (ROIs). Time courses depicting bilateral (Left = Left Hemisphere, Right = Right Hemisphere) activation (measured by dSPM values) for both exposure durations (blue = short, red = long) and both threat cue types (brighter shades = averted gaze [“clear” threat], darker shades = direct gaze [“ambiguous” threat]) in our Regions of Interest (ROIs). Standard Error from the Mean (SEM) is represented by dashed lines of the same color above and below the respective time series. The dashed black line indicates stimulus offset for brief exposures (250 ms) while the solid black line indicates stimulus offset for long exposures (883 ms).

The time courses of activation within our ROIs revealed an effect of stimulus exposure duration for both averted-gaze fear and direct-gaze fear. The time courses can be seen in Fig. 3, and a comprehensive list of the timing and significance of these effects can be found in Table 4. In V1 and FFA, we found significantly increased activation for brief compared to longer exposures only in the right hemisphere and only for averted-gaze fear for V1 (*p* = 0.02) and direct-gaze fear for FFA (*p* = 0.04). Conversely, we observed significantly increased activation from longer presentations over brief presentations bilaterally for both gaze directions in V1 and FFA. Bilateral V1 and right FFA were the only regions in which we observed significantly greater activity from the longer-exposure condition, while the stimulus was still on the screen, compared to the brief-exposure condition after stimulus offset (Fig. 3). In pSTS we observed a similar temporal progression, but found significant differences in evoked activity between exposure durations only during the viewing of direct-gaze fear faces (see Table 3 for timing and significance). PAC and OFC bilaterally demonstrated a distinctly different temporal progression of activity compared to the other regions (Fig. 3). This was characterized in the brief-exposure condition by a rise in activity starting around 500 ms, hundreds of milliseconds after the stimulus offset at 250 ms. This increase superseded activity in the longer-exposure condition at around 600 ms, even though the stimulus was still being displayed (see Fig. 3 and Table 4 for the timing and statistics of significant differences). This activity in response to the brief-exposure stimulus remained stronger until around 1000 ms (750 ms after stimulus offset) when a similar pattern began to emerge from the longer exposure, evoked by the removal of the threat cue. Thus, in pSTS, PAC, and OFC, the activity after the stimulus offset (post-250 ms) in the brief-exposure condition was higher, and showed a different pattern, than activity in the longer-exposure condition in which the stimulus was still present (250–883 ms).

**Table 4:** Timing of significant differences in activation within ROIs between brief (250 ms) and long (883 ms) exposures (reported *p*-values are non-parametric and corrected based on cluster permutations thresholded at *p* < 0.05 uncorrected).

ROI	LH		RH	
	Time (ms)	p-value	Time (ms)	p-value
**Averted Gaze**				
*Brief minus Long*				
V1	n.s.	n.s.	150–220 320–365	0.01 0.02
FFA	n.s.	n.s.	n.s	n.s
pSTS	n.s	n.s	n.s	n.s
PAC	n.s.	n.s.	620–820	0.001
OFC	600–800	0.02	600–820	0.01
*Long minus Brief*				
V1	500–1300	0.0004	420–1300	0.0002
FFA	900–1300	0.0004	800–1300	0.0002
pSTS	n.s	n.s	n.s	n.s
PAC	1020–1300	0.002	1100–1300	0.0006
OFC	1075–1300	0.004	1100–1300	0.002
**Direct Gaze**				
*Brief minus Long*				
V1	n.s.	ns.	n.s.	n.s
FFA	n.s.	n.s.	300–380	0.04
PSTS	380–480	0.05	n.s.	n.s.
PAC	n.s.	n.s.	620–780	0.03
OFC	n.s.	n.s.	620–800	0.02
*Long minus Brief*				
V1	500–1300	0.0002	400–1300	0.0002
FFA	900–1080	0.02	800–1300	0.0002
pSTS	975–1300	0.003	950–1100	0.04
PAC	1100–1300	0.03	1100–1300	0.001
OFC	1050–1300	0.01	1050–1300	0.0008

In summary, effects of exposure duration dominated the activity in all ROIs we examined. The nature of these effects seemed to depend on the role of the region in the processing hierarchy, as PAC and OFC, showed strongest responses after stimulus offset. To examine these effects in greater detail, we performed phase-locking analyses in all the ROIs for frequencies in the α and β bands.

#### Effects of Exposure Duration: Phase Locking

**Figure 4:**
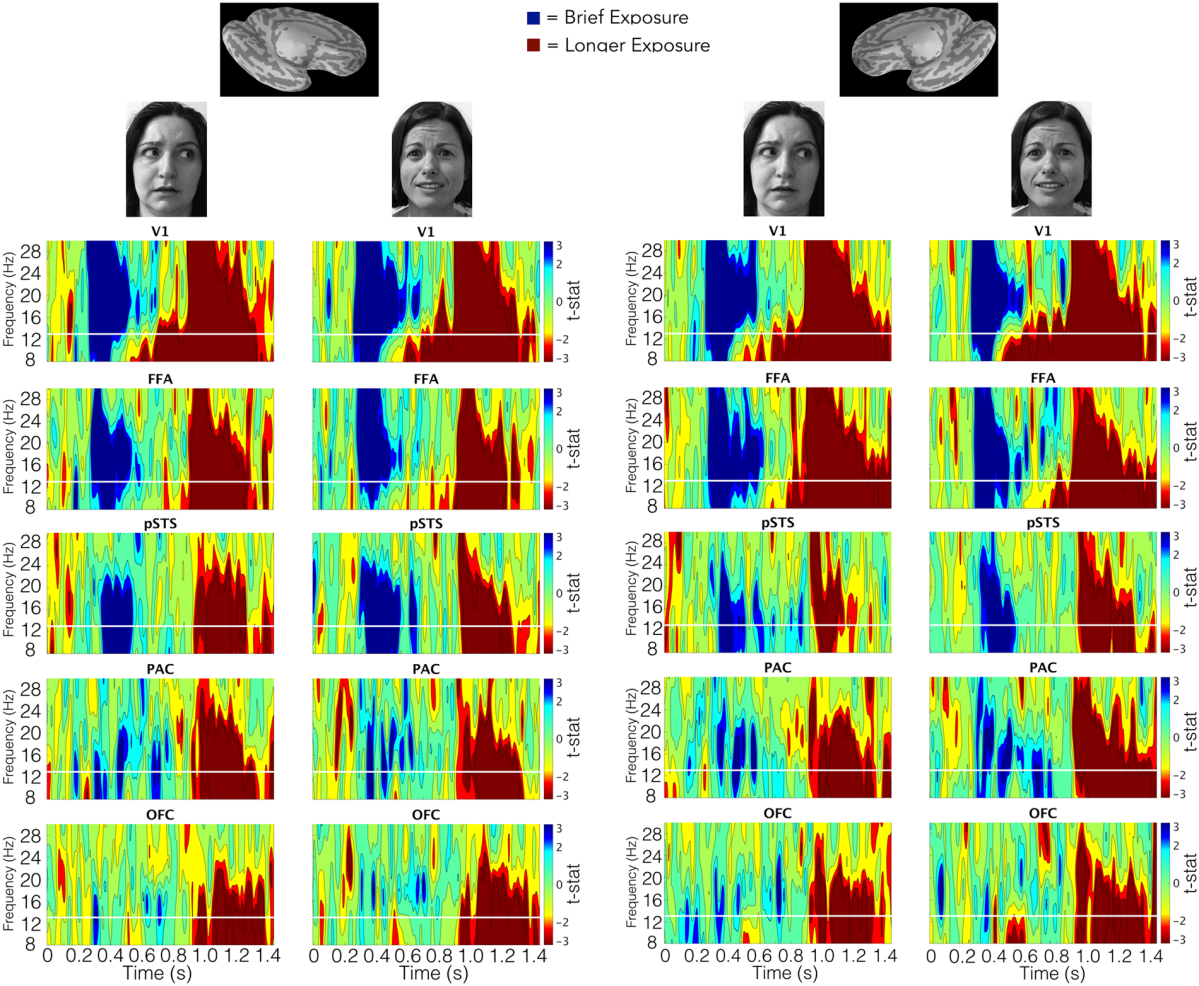
Unthresholded statistical maps of brief -versus longer-exposure phase locking across trials by threat cue type. The left side shows maps for each ROI from the left hemisphere according to the threat type conveyed by the face at the top of the column (averted-gaze = clear threat, direct-gaze = ambiguous threat). The right side shows the same for the right hemisphere. For each map, the y-axis represents frequency (in Hz) while the x-axis represents time (in s) while the pixel value is the t-statistic representing each participant’s PLF (Phase-locking Factor) across trials for brief exposures (represented in blue) compared to long exposures (represented in red). Contour levels map to significance based on a two-tailed distribution with 59 degrees of freedom. Green represents no significance (i.e. *p-*values above 0.05). The three blue shades (cyan, blue, dark/navy blue) represent p-value ranges between 0.05–0.01, 0.01–0.001, and 0.001 and below for brief exposures. The three red shades (yellow, red, dark red) represent the same p-values for long exposures. All *p*-values parametric and uncorrected.

Examination of phase synchrony across trials in our ROIs also yielded powerful effects of stimulus exposure duration for both averted-gaze and direct-gaze fear (Fig. 4). See Tables 5 and 6 for the exact timing and significance of these effects in the α and β frequency ranges, respectively. Similar to the activity we observed in the time courses, there were effects of exposure duration in every ROI examined. Phase-locking exposure effects were quite robust as the vast majority of them corresponded to a non-parametric *p* value of 0 (i.e., not a single randomly shuffled data set in the permutation process out of 5000 yielded a cluster larger than that which was observed in the actual data). V1, FFA, and pSTS bilaterally all had significantly greater phase locking to both brief and longer exposures within the trial in both α and β frequency bands (Fig. 4). The results mirrored those in the time course analyses, in that the phase locking in response to the brief-exposure condition was greater when the stimulus was already off the screen, compared to the longer-exposure condition. The early visual and face-processing regions V1 and FFA were the only regions to show significantly greater phase locking to the longer exposure before the longer-exposure stimulus was removed from the screen (Fig. 4). For PAC, we observed significantly greater phase locking to both stimulus exposures for both threat types in the β band. However, activity in the α band was sensitive primarily to longer exposures, with the exception of direct gaze in the left hemisphere, which evoked responses to both the brief and longer-exposure conditions. OFC was engaged primarily by the longer-exposures trials in both bands. The only exception was in left OFC, which displayed stronger β phase locking to the brief exposure during direct-gaze threat cue viewing (*p* = 0.05).

**Table 5:** Timing of significant differences in α phase locking across trials within ROIs between brief (250 ms) and long (883 ms) exposures (reported *p*-values are non-parametric and corrected based on cluster permutations thresholded at *p* < 0.05 uncorrected).

ROI	LH		RH	
	Time (ms)	p-value	Time (ms)	p-value
**Averted Gaze**				
*Brief minus Long*				
V1	320–560	0.007	310–520	0.01
FFA	320–600	0.004	300–660	0.002
pSTS	400–610	0.0006	390–580 820–940	0.002 0.05
PAC	n.s.	n.s.	n.s.	n.s.
OFC	n.s.	n.s.	n.s.	n.s.
*Long minus Brief*				
V1	570–1440	0	730–1440	0
FFA	900–1400	0	820–1440	0
pSTS	990–1340 1370–1440	0 0.02	1010–1200	0.009
PAC	980–1420	0	975–1400	0
OFC	980–1400	0.0008	975–1400	0.0002
**Direct Gaze**				
*Brief minus Long*				
V1	310–570	0.006	320–400	0.01
FFA	350–560	0.03	300–500	0.001
PSTS	380–630	0	380–620	0
PAC	n.s.	n.s.	620–870	0.01
OFC	n.s.	n.s.	n.s.	n.s.
*Long minus Brief*				
V1	590–1440	0	480–1440	0
FFA	940–1300	0	820–1440	0
pSTS	1000–1350	0	1000–1360	0
PAC	970–1400	0	980–1440	0
OFC	980–1330	0	1080–1440	0.0002

#### Effects of Gaze: Time Courses

Permutation cluster tests of the time courses within our ROIs revealed only one effect of gaze. We observed significantly increased activation from direct-gaze threat cues during longer exposures in V1 of the right hemisphere late in the trial around 500–650 ms (*p* = 0.02). Given its timing, this effect may be the result of feedback from higher regions.

#### Effects of Gaze: Phase Locking

**Figure 5:**
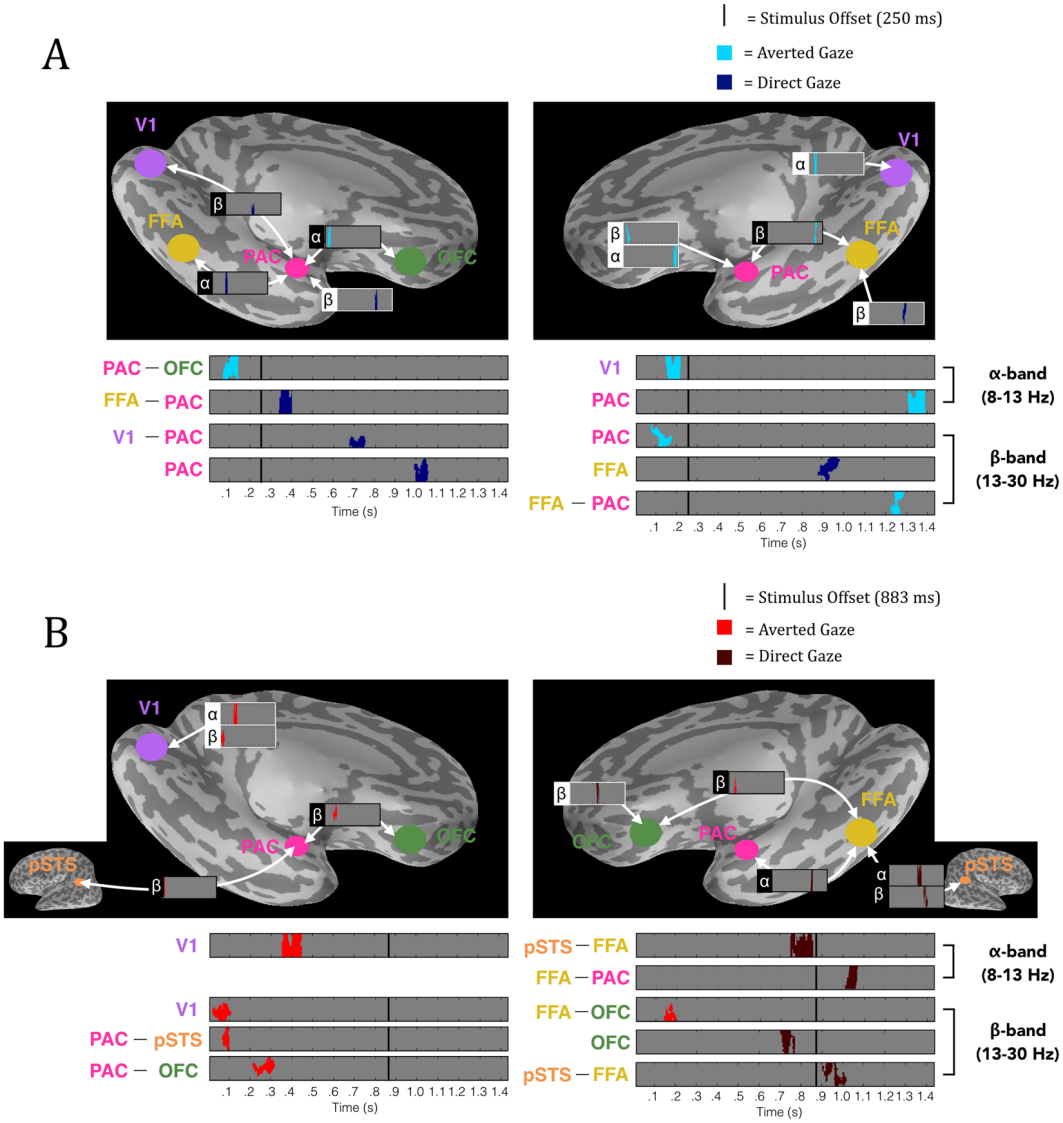
Significant phase locking for averted vs. direct gaze fearful faces in the extended face-processing network. (**A**) Significant phase locking for averted-gaze (bright blue) vs. direct-gaze (dark blue) fearful faces during brief exposures (250 ms). (**B**) Significant phase locking for averted gaze (bright red) vs. direct gaze (dark red) fearful faces during long exposures (883 ms). *Left* is the left hemisphere phase-locking network (denoted by the image of the left hemisphere). *Right* is the right hemisphere phase-locking network. *Above* is the significant phase locking overlaid on an inflated *Freesurfer* brain to show anatomical locations of significant phase locking. White outlines depict phase locking (PLF) within a region (i.e. phase locking across trials) while black outlines depict phase-locking values (PLV) between regions. The phase locking’s frequency band is marked by the appropriate Greek letter to the left (α = 8–13 Hz, β = 13–30 Hz). *Below* is the significant phase locking magnified and arranged by time of effects as well as frequency band of effects (α on top, β on bottom, as noted on the far right) to display the temporal progression of significant phase locking through the trial. The anatomical location/connection of the phase locking is noted to the left. Stimulus offset is marked by the solid black line. For all plots, grey depicts periods of non-significant phase locking while colored (see legend/A,B descriptions for color to trial type correspondence) blobs depict significant phase locking determined by non-parametric cluster permutation tests as outlined in *Methods*.

*Brief Stimulus Exposures (250 ms)*:

*Significant phase-locking differences for averted-gaze fear faces.* The first effect we observed was an increased bilateral response to averted gaze over direct-gaze fear faces. Averted-gaze fear evoked stronger phase locking in the *α*-band early between left PAC and OFC (*p* = 0.013) around 80–160 ms and in right V1 (*p* = 0.041) around 140–220 ms (Fig. 5A). We also found significantly stronger phase locking for averted-gaze compared to direct-gaze fear in the β-band of right PAC at around 120 ms (*p* = 0.023). These findings support our hypothesis of initial reflexive processing being more sensitive to averted-gaze fear, since these congruent cues are thought to be processed more efficiently^15^. In addition, we found stronger phase locking for averted-gaze fear compared to direct-gaze fear very late at the end of the brief-exposure trial. This occurred in the β-band between right FFA and PAC around 1200–1300 ms (*p* = 0.012) as well as in the α-band of right PAC from approximately 1320–1380 ms (*p* = 0.035) (Fig. 5A). Right PAC’s stronger response to averted gaze during brief exposures is in line with our fMRI findings in the right amygdala, which showed greater activation to averted-gaze fear faces compared to direct-gaze fear faces during brief exposures (Fig. 5A).

Overall, we found two distinct time windows during the brief-exposure trials in which phase locking, both across trials within a region and in the connectivity between regions, was significantly stronger when exposed to averted-gaze fear faces compared to direct-gaze fear faces: A bilateral response early in the trial around 80–220 ms and then a late response concentrated in the right hemisphere around PAC past 1200 ms (Fig. 5A).

*Significant phase-locking differences for direct-gaze fear faces*. For direct-gaze fear faces, there was significantly increased phase locking compared to averted-gaze fear faces during the mid-trial period. We found significantly increased phase locking compared to averted-gaze fear faces in the α-band between left FFA and PAC (*p* = 0.04), peaking at around 350 ms. This occurred following the stimulus offset at 250 ms, possibly suggesting that removal of the direct-gaze fear face (incongruent threat cue) elicits sensitivity to this type of threat cue (Fig. 5A). Phase locking continued to be stronger to direct-gaze fear in the β-band between left V1 and PAC around 700 ms (*p* = 0.001), in the right FFA around 900 ms (*p* = 0.05), and in left PAC around 1000 ms (*p* = 0.03). Thus, with brief exposures, there is a relative increase of phase locking to direct-gaze fear compared to averted-gaze fear faces beginning immediately after removal of the stimulus up until around 1050 ms.

In summary, during brief exposures we found stronger early *and* late phase locking to averted-gaze fear faces, and stronger phase locking to direct-gaze fear faces mid-trial around the 300–1050 ms range, with right PAC responding more to averted-gaze fear and left PAC responding more to direct-gaze fear. These MEG findings are broadly consistent with the fMRI findings in the amygdalae using this paradigm (^9,15,54^, Experiment 1 in this study).

*Longer Stimulus Exposures (883 ms)*: *Significant phase-locking differences for averted-gaze fear faces*. With exposures of 883 ms, we likewise observed greater β-band phase locking in response to averted-gaze fear faces early in the trial: in left V1 around 60–80 ms (*p* = 0.017), between left PAC and pSTS around 80–100 ms (*p* = 0.032), between right FFA and OFC around 140–200 ms (*p* = 0.033), and between left PAC and OFC (*p* = 0.003) at approximately 200–300 ms (Fig. 5B). This again demonstrates a reflexive response more tuned to averted-gaze fear immediately following exposure to the threat cue, suggesting it to be the more salient threat cue to the face processing network. Averted-gaze fear faces also evoked stronger α-band phase locking in left V1 (*p* = 0.042) at around 400 ms (Fig. 5B) compared to direct-gaze fear. In contrast to brief exposures, stronger phase locking for averted-gaze fear face stimuli persisted until 400 ms suggesting that greater averted-gaze fear phase locking early in the trial is not inherently limited to the pre-300 ms early time frame but can be more sustained when the stimulus exposure is longer. That is, the exposure duration modulates the initial response to averted-gaze fear faces, with longer exposures resulting in longer processing times for averted-gaze fear, when compared to the processing of direct-gaze fear faces under the same exposure conditions.

*Significant phase-locking differences for direct-gaze fear faces*. Towards the latter half of the trial, phase locking became stronger to fearful faces with direct gaze relative to averted gaze in the α-band between pSTS and FFA around 700–950 ms (*p* = 0.005) and between FFA and PAC between 1000–1100 ms (*p* = 0.034), as shown in Fig. 5B. During this same time frame, phase locking in the β-band to direct-gaze fear faces significantly increased over averted-gaze fear faces in right OFC around 700–800 ms (*p* = 0.034) and between right pSTS and FFA at roughly 900–1000 ms (*p* = 0.01). This provides evidence for our hypothesis that longer presentations result in more extensive processing of direct-gaze fear faces, as all of the significant phase-locking activity differences demonstrated higher phase locking for direct-gaze fear faces late in the trial for connections in the right hemisphere between PAC, FFA, OFC and pSTS.

To conclude, during longer exposures we see similar stronger early phase locking for averted-gaze fear faces relative to direct gaze as we observed in in the brief-exposure trials. However, longer stimulus exposures also prolong this initial processing of averted-gaze fear faces, and delay and prolong processing of direct-gaze fear faces. Returning to greater phase locking for averted-gaze fear faces very late in the trial occurred only during the brief-exposure trials.

## Discussion

The primary aim of the present study was to characterize the neurodynamics mediating facial threat cue perception with MEG, building on previous fMRI findings that employed the same stimulus set of congruent (fearful faces with averted eye gaze) or incongruent (fear with direct gaze) cues^15^. We first validated our experimental design as a direct comparison to previous efforts by replicating the findings of block-design experiments^9,14,15^ using an event-related paradigm in fMRI. In Experiment 1 (fMRI), we replicated the findings of Adams *et al*.^15^, finding that amygdala responses vary as a function of stimulus exposure duration in response to averted-gaze and direct-gaze fear faces. BOLD activity in the right amygdala was enhanced to averted-gaze fear during brief exposures and direct-gaze fear during longer exposures, whereas the left amygdala activity was greater for longer-exposure durations, particularly for direct-gaze fear. These fMRI findings suggest both that brief stimulus exposures elicit a stronger reflexive response to averted-gaze fear, and that longer exposures evoke more reflective processing of direct-gaze fear, possibly in order to resolve the incongruity in the latter cue combination. These findings align with previous demonstrations of the amygdala being sensitive to brief exposures of averted-gaze fear faces^9,85^ as well as longer exposures of the incongruent threat cues conveyed by direct-gaze fear^14,86^. However, because of the temporal limitations of fMRI and the BOLD signal, they offer only a partial description of how facial fear cues are processed in the brain, as we detail below.

In Experiment 2 (MEG), we aimed to characterize the effects of exposure duration and compound facial threat cues on the neurodynamics of threat perception on a finer temporal scale by examining the time courses and phase locking of activity in the extended face network, including FFA, PAC (the periamygdaloid cortex), pSTS, and OFC, as well as the primary visual cortex. We observed robust effects of presentation duration in all the ROIs we examined. Brief stimulus exposures for both direct and averted-gaze fear faces resulted in stronger activity immediately following stimulus offset, compared with activity when the stimulus was still present during the longer-exposure trials. In PAC and OFC, this activity persisted for hundreds of milliseconds after the stimulus had been removed in a slow second cycle of processing. Conversely, the longer stimulus exposures elicited significantly greater activation from all our ROIs during the mid-to-late trial period. This would suggest that there is indeed an inherent difference in how these areas respond to facial threat cues, driven by the exposure duration.

We expected brief stimulus presentations to elicit more reflexive threat processing (see^16–18^), and thus a greater response to averted-gaze fear, which previously had been found to be processed more quickly and efficiently than direct-gaze fear (e.g.,^8,15^). Examination of the phase locking in and between our ROIs indeed revealed a greater initial response to averted-gaze fear in both the α and β frequency ranges. Because of the primary focus on the response in the amygdala complex in previous fMRI studies^9,14,15,54^, it should be emphasized that we observed stronger phase locking to averted-gaze fear in the initial response of the right PAC, as well as in the initial connectivity between left PAC and OFC, suggesting more preconscious attention to congruent threat cues in these regions. This adds to the growing body of neuroimaging evidence that fear is processed quickly by the amygdaloid complex with the help of surrounding connected regions^87–90^. It is worth noting that these early differentiations between congruent and incongruent threat cues either temporally paralleled or preceded similar responses in the early visual cortex (V1) leaving open the possibility of an alternate route to the amygdala via subcortical projections, circumventing V1 and typical initial visual processing^90,91^. However, increased phase locking between PAC and OFC during this early response indicates it must involve more than subcortical connections. Previous work has shown that rapid magnocellular projections to the OFC enable top-down facilitation of bottom-up processes such as object recognition^62^. Due to the extensive connections between the amygdala and OFC it is conceivable that similar rapid projections to the OFC are active during facial threat perception, which could then facilitate bottom-up amygdala processing of clear threat messages (congruent threat cues). Additionally, this finding is consistent with the behavioral findings of Milders *et al*.^8^ and Adams’ shared signal hypothesis^4,14,15^, suggesting averted-gaze fear is the more salient threat cue. Immediate sensitivity to the more salient threat cue in right PAC is consistent with the right amygdala’s putative function in rapid detection of emotionally salient stimuli^92^.

Once the stimulus was removed at 250 ms in the brief presentation condition, we found that phase locking then increased in response to direct-gaze fear faces from about 300–1000 ms, and then was superseded by averted-gaze fear-related activity very late in the trial. Generally, left PAC (along with the left hemisphere as a whole) showed more sensitivity to direct-gaze fear faces during the brief exposures. This is also congruent with the left amygdala’s putative involvement in reflective assessment of stimuli following an initial, reflexive emotional response from the right side^92^. The stronger phase locking to direct-gaze fear during this period reveals a more nuanced picture of PAC activity than that suggested by fMRI results in the amygdala: namely, that the amygdaloid complex appears to be sensitive to threat cues portrayed by both averted and direct-gaze fear at different times. Therefore, the greater BOLD signal evoked by averted-gaze fear during brief exposures in fMRI, as in Experiment 1 and in previous fMRI studies^9,15^ is likely due to the nature of fMRI; that is, summation of (delayed and temporally dilated) BOLD signal, which is unable to resolve fine-grained sensitivity of the amygdala complex to different threat cues at different stages of processing. These results might be partially explained by our finding in MEG that, at the end of the trial (1100–1400 ms), phase locking to congruent threat cues again became stronger than for incongruent threat cues. Significant differences this late in the trial are far too late to be considered “reflexive”, indicating that there is more at play in the neurodynamics mediating threat processing than just an automatic, reflexive response to the brief exposure, followed by a reflective response if stimulus presentation is maintained.

Based on the fMRI findings using this paradigm^9,14,15,54^, we had predicted that longer stimulus exposures would evoke a stronger late response to direct-gaze fear, possibly indicative of reflective analysis to resolve ambiguity in these incongruent threat cues. However, because the brief and longer presentation conditions are identical for the first 250 ms, phase locking in and between our ROIs again showed a stronger initial response to averted-gaze fear in both the α and β frequency bands, which resembled the response during the brief stimulus exposures. However, this stronger phase locking to averted gaze was sustained longer, compared with the brief-exposure condition, and extended past the point at which direct-gaze fear-related processing had superseded averted-gaze threat-related processing in the brief duration trials. Similar to brief exposures, mid-trial phase locking during the longer-exposure trials was stronger to direct-gaze fear faces compared to averted-gaze fear faces, but this sensitivity to threat signal incongruity began later and was not superseded by increased phase locking to congruent threat cues later in the trial, as in the brief-exposure trials.

In addition to the temporal differences, we found differences in the spatial pattern of significant phase locking. Compared to the phase locking during brief-exposure trials, which was concentrated around left PAC, phase locking during longer exposures was centered mostly around right FFA. Reflective processing is associated more with the ventral stream^16^, of which the FFA is a key module. Thus, greater involvement of FFA during longer-exposure trials may provide some support to our hypothesis of reflective processing being biased toward ventral stream processing. A notable connection for right FFA during longer exposures was to right pSTS, which displayed two temporally and spectrally distinct periods of incongruent threat-cue sensitivity immediately preceding and following stimulus offset. The late involvement of pSTS during longer exposures could potentially be representative of its function as part of the “social brain”^93^, as the right pSTS in particular has been linked to internalizing another’s perspective^94^. It is thought to be a key region for visual integration of social cues, especially when inferring social meaning from combinations of facial expression and eye gaze^95^, making it a sensible candidate to be recruited in resolving possible ambiguity of the incongruent threat cue during longer exposures. Collectively, the phase locking seen in the right hemisphere supports the idea of slower reflective analysis being mediated by the ‘high road’^85^ during longer exposures to resolve the meaning of the incongruent threat cue. This appears at odds with the left hemisphere’s role as being primarily responsible for reflective analysis in previous findings^96^. One speculative resolution of this incongruity could lie in the general right hemisphere specialization for face processing^97,98^ as well as in the right STS’s specialization in gaze perception^99^. The nature of such late activity is difficult to interpret, however, particularly since most studies on the topic have been done with fMRI and were not explicitly designed to test the lateralization of the amygdala responses. It is worth noting that there is growing evidence for sex differences in processing emotional stimuli^100^. Indeed, other recent work has shown sex differences in amygdala lateralization in response to congruent and incongruent facial fear and eye gaze cues with fMRI^101^. Unfortunately, the current fMRI data cannot offer a clear conclusion on the sex-related differences due to the highly unequal and small sample size, especially for male participants (N = 4). However, does offer a direct replication of a previous fMRI study by our group (Adams *et al*.^15^) in which the sample size was more balanced across the sexes (15 females to 14 males). To verify that our results here cannot be explained by sex-related differences, we ran separate non-parametric cluster-based permutation tests based on an independent sample *t-*test for significant differences in PAC phase locking between males (N = 20) and females (N = 40). No significant differences were found between males and females in any of our 4 conditions in either hemisphere (all *p*’s > 0.24), suggesting our results were not driven by sex-related effects.

In our phase-locking results, we did not see much evidence for functional separation between the α and β bands. However, compared to other frequency bands, α-band activity does not fit into a general hypothesis of function^102,103^. In the specific context of α activity evoked by viewing emotional faces, α responses have been shown to be higher in response to negative (angry) compared to positive (happy) emotional faces over temporal, parietal, and occipital recording sites^104^, but weaker in response to valenced faces (both positive and negative) compared to neutral faces^105,106^. The evidence for any specific α-band function in emotional perception is sparse and inconsistent, but none of it suggests the role of inhibition^103^. We found α-band activity to be sensitive to negative valence, as α phase locking was stronger both in initial reflexive processing of averted-gaze fear, suggesting a role in increasing shared-signal processing efficiency, and in late reflective processing of the direct-gaze fear (incongruent signal). In addition, α-phase locking was mostly congruent with β activity both in effects of stimulus duration and effects of eye-gaze, supporting an active non-inhibitory role for α-band oscillations during facial threat cue perception, at least in terms of its phase locking (compared to power or other magnitude-dependent measures).

An important limitation of the current work reported here is that we only examined neurodynamics in response to congruent and incongruent threat cues portrayed by fearful faces. Future work will be necessary to supply additional evidence that threat cue congruency is the primary driving factor behind these results, a situation similar to the previous works upon which these studies are based^9,14,15,54^. In addition, we had no evidence from the previous fMRI studies that identical timing parameters would apply to threat cues portrayed by angry faces compared to fearful faces. However, initial efforts with longer exposures do suggest these effects to not simply be an effect of gaze as the original work demonstrated that incongruent cues (direct-gaze fear and averted-gaze anger) elicited stronger amygdala activation than their congruent counterparts despite these cues featuring opposite gaze directions. To provide categorical support for the shared signal hypothesis, future time-resolved explorations will be necessary utilizing other types of congruent and incongruent threat cues, such as anger faces.

In summary, with fMRI, we replicated previous stimulus exposure duration effects for fear expression processing as a function of gaze perception using a broad stimulus set and employing an event-related design, and showing that previous findings using this paradigm^9,14,15,54^ are not dependent upon states induced by a block design. This set the stage for using this identical paradigm in MEG to examine the temporal dynamics of the effect. We found that the stimulus exposure duration strongly modulated the fine-grained neurodynamics in the extended face perception network, resulting in a slower processing sequence for the longer-exposure stimuli. However, the effects of gaze, while modulated by the exposure duration, were nonetheless evident in greater early phase locking to averted gaze, providing neural evidence at a high temporal resolution that these congruent compound cues are indeed allocated more preconscious attention, and in increased later phase locking to direct gaze, again supporting the idea that slow, reflective processing is biased towards the incongruent threat cue. However, the fast neurodynamics in the extended face-processing network in response to these brief and longer exposures observed with MEG show that its response is far more complicated than a simple dichotomy of a reflexive response induced by a brief stimulus exposure and a reflective response induced by a longer stimulus presentation. Here we have shown that the observed greater BOLD response to averted-gaze fear in the amygdala complex during brief exposures is not the result of the processing being curtailed by the brief presentation, and thus cutting off reflective processing. Rather, it likely stems from the summation of the hemodynamic signal over the entire trial in fMRI, as we observed stronger mid-trial phase locking to direct-gaze fear during both brief and longer exposures, and the initial reflexive response to averted-gaze fear, again regardless of exposure duration. Previous attempts to rationalize the opposing findings in fMRI suggested that congruent cues such as averted-gaze fear not only capture attention more readily, but due to their aversive nature, may result in more rapid disengagement, explaining the comparative dwelling of the neural response on direct-gaze fear faces during longer exposures^107^. The ability to empirically address this speculation with the high temporal resolution of MEG revealed that this does not in fact seem to be the case, as we observed prolonged cognitive engagement with averted-gaze fear relative to direct-gaze fear under longer exposures. Our findings thus underscore the importance of investigating brain activity using techniques with high temporal resolution to gain a more complete picture of the neurodynamics underlying perception of compound threat cues from the face.

**Table 6:** Timing of significant differences in β phase locking across trials within ROIs between brief (250 ms) and long (883 ms) exposures (reported *p*-values are non-parametric and corrected based on cluster permutations thresholded at *p* < 0.05 uncorrected).

ROI	LH		RH	
	Time (ms)	p-value	Time (ms)	p-value
**Averted Gaze**				
*Brief minus Long*				
V1	300–690	0	290–650	0
FFA	320–680	0	320–690	0
pSTS	380–630	0	390–580	0.0002
PAC	490–700	0.005	350–570	0.002
OFC	n.s.	n.s.	n.s.	n.s.
*Long minus Brief*				
V1	750–1400	0	830–1440	0
FFA	950–1350	0	920–1440	0
pSTS	980–1330	0	980–1200	0
PAC	970–1390	0	980–1200	0
OFC	1100–1420	0	980–1090 1100–1400	0.007 0
**Direct Gaze**				
*Brief minus Long*				
V1	310–760	0	320–660	0
FFA	320–640	0	330–680 760–900	0 0.04
PSTS	640–640	0	360–620	0
PAC	340–460 480–710	0.01 0.0002	340–480 480–790	0.001 0.001
OFC	630–790	0.05	n.s	n.s.
*Long minus Brief*				
V1	820–1370	0	830–1420	0
FFA	970–1300	0	950–1420	0
pSTS	980–1330	0	990–1360	0
PAC	970–1390	0	960–1440	0
OFC	1080–1400	0	980–1090 1090–1440	0.003 0
